# Using Ribosomal Protein Genes as Reference: A Tale of Caution

**DOI:** 10.1371/journal.pone.0001854

**Published:** 2008-03-26

**Authors:** Lieven Thorrez, Katrijn Van Deun, Léon-Charles Tranchevent, Leentje Van Lommel, Kristof Engelen, Kathleen Marchal, Yves Moreau, Iven Van Mechelen, Frans Schuit

**Affiliations:** 1 Gene Expression Unit, Department of Molecular Cell Biology, Katholieke Universiteit Leuven, Leuven, Belgium; 2 Department of Electrical Engineering, ESAT-SCD, Katholieke Universiteit Leuven, Leuven, Belgium; 3 Department of Psychology, Katholieke Universiteit Leuven, Leuven, Belgium; 4 Department of Microbial and Molecular Systems, Katholieke Universiteit Leuven, Leuven, Belgium; 5 SymBioSys, K.U.Leuven Center for Computational Systems Biology, Leuven, Belgium; Children's Hospital Boston, United States of America

## Abstract

**Background:**

Housekeeping genes are needed in every tissue as their expression is required for survival, integrity or duplication of every cell. Housekeeping genes commonly have been used as reference genes to normalize gene expression data, the underlying assumption being that they are expressed in every cell type at approximately the same level. Often, the terms “reference genes” and “housekeeping genes” are used interchangeably. In this paper, we would like to distinguish between these terms. Consensus is growing that housekeeping genes which have traditionally been used to normalize gene expression data are not good reference genes. Recently, ribosomal protein genes have been suggested as reference genes based on a meta-analysis of publicly available microarray data.

**Methodology/Principal Findings:**

We have applied several statistical tools on a dataset of 70 microarrays representing 22 different tissues, to assess and visualize expression stability of ribosomal protein genes. We confirmed the housekeeping status of these genes, but further estimated expression stability across tissues in order to assess their potential as reference genes. One- and two-way ANOVA revealed that all ribosomal protein genes have significant expression variation across tissues and exhibit tissue-dependent expression behavior as a group. Via multidimensional unfolding analysis, we visualized this tissue-dependency. In addition, we explored mechanisms that may cause tissue dependent effects of individual ribosomal protein genes.

**Conclusions/Significance:**

Here we provide statistical and biological evidence that ribosomal protein genes exhibit important tissue-dependent variation in mRNA expression. Though these genes are most stably expressed of all investigated genes in a meta-analysis they cannot be considered true reference genes.

## Introduction

A challenge for the accurate quantification of differences in gene expression level across biological conditions is to normalize for potential artifacts caused by sample preparation or gene expression detection. A common technique in RT-PCR, northern blots or western blots is to normalize data for such artifacts by measuring in the same samples the expression of a reference gene in parallel. The reference gene(s) are assumed to be expressed at constant levels across all the experimental conditions, tissues or cell lines. When only one tissue or cell line is studied, it suffices to look at genes that are constantly expressed in that particular tissue, but need not be expressed in other tissues. In the study of the relative levels of gene expression in various tissues, such as in the study of tissue-specific regulatory elements, a gene that is expressed at constant levels in many tissues is needed. The choice for such reference gene(s) has been a subject of debate for many years. Typical choices were beta-actin, *GAPDH, HPRT*, or *18S RNA*. These genes were thought to be stably expressed since they are considered as “housekeeping genes”. Housekeeping genes have been defined functionally as “constitutively expressed to maintain cellular function” [1]. Being constitutively expressed however does not necessarily meet the prerequisites for a good reference gene, which also needs to display a sufficiently small variation in expression among different tissues. Many of the commonly used reference genes exhibit considerable variability in expression over different tissues and/or experimental conditions [2]–[5] and therefore are not a good choice as reference. More recently, several attempts were performed based on microarray or large-scale sequencing technologies to find more stably expressed reference genes. A meta-analysis of 13629 human Affymetrix arrays was conducted to identify the most stably expressed genes [6]. As a result, a list of 15 genes was suggested with the most constant expression level, based on a coefficient of variation smaller than 4%, a maximum fold change smaller than 2 and a mean expression level lower than the maximum expression level minus 2 times the standard deviation. Thirteen of these fifteen genes are coding for ribosomal proteins. We assessed the expression stability of ribosomal proteins across 22 tissues and suggest that, from a biological and statistical perspective, one should be careful to use these genes as reference genes.

## Results

### 1. Ribosomal proteins genes: housekeeping but also reference?

#### 1.1 Ribosomal genes are constitutively expressed

**Figure 1:**
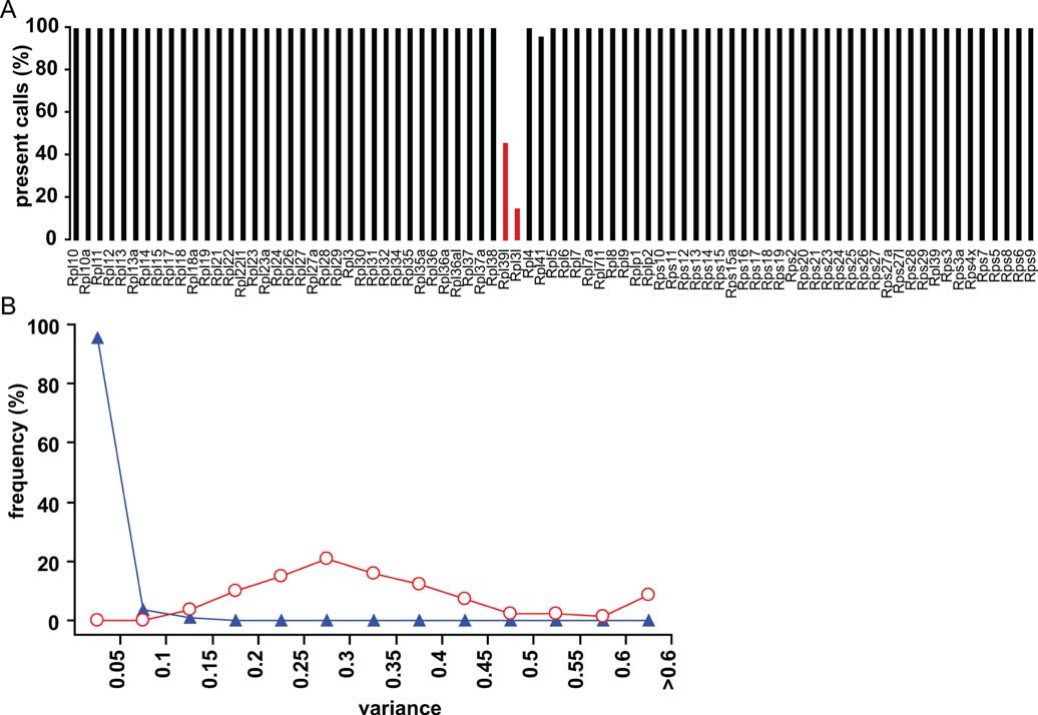
Housekeeping versus reference gene status of 81 ribosomal protein genes. (A) Percentage of present expression calls tested in 22 different mouse tissues (3–5 replicates per tissues, in total 70 arrays). Most ribosomal protein genes were present in all tissues examined and thus can be called housekeeping genes. Exceptions were *Rpl39l* and *Rpl3l*. (B) Variance of expression levels within replicates of tissues, representing biological variation between animals and technical error on measurements (triangles) compared to variance of expression between tissues (circles). Variance within replicated measurements was significantly smaller than variance of expression between different conditions (Wilcoxon's rank sum statistic: p<0.0001).

It was suggested before [7] that ribosomal genes are good housekeeping genes as they are expressed in all cell types to direct biogenesis of new ribosomes. To validate this, we analyzed the probesets for 81 different ribosomal protein genes represented on the Affymetrix mouse 430 2.0 expression arrays in a set of 22 different mouse tissues. In total, 6951 probesets were called present over all individual arrays which was 15% of all probesets on the 430 2.0 array and corresponded to 4845 unique genes (20% of estimated number of mouse genes). With a few exceptions, all ribosomal protein mRNAs were called present over all individual arrays and two were exceptionally called absent (Figure 1A). Transcripts from these two genes, *Rpl39*-like and *Rpl3*-like, were only expressed in a few tissues. Therefore, based on their ubiquitous expression profiles, we could endorse the status of most ribosomal protein genes as housekeeping genes. The next question arising was whether these genes would be suitable as reference genes.

#### 1.2 Variation analysis within versus between different tissues

For all 81 probes representing the ribosomal protein genes, we found with one-way analysis of variance (ANOVA) that the expression levels differed significantly between tissues at a simultaneous significance level of .01 (using a Bonferroni correction to account for multiple testing). To get a clearer picture of the different sources of variation, we performed a two-way ANOVA with the 22 tissues and the 81 probesets representing ribosomal protein genes considered as the factors of variation. The gene effect was most significant, reflecting different average expression levels of ribosomal protein genes over all tissues (F_80,5669_ = 5598.61, p<0.0001). After correcting for gene variation, the tissue effect was also highly significant (F_21,5669_ = 4094.43, p<0.0001), reflecting that in general ribosomal proteins as a group were more highly expressed in certain tissues. In addition to these main effects (genes as a group or tissues as a group), significant variation could be attributed to the gene-tissue interaction effect (F_1680,5669_ = 38.55, p<0.0001), reflecting gene specific deviations in expression across various tissues.

In order to assess the importance of expression variation of the ribosomal protein genes among different mouse tissues, we first compared the variance amongst replicates of the same tissue from different animals (representing technical variation plus inter-individual variation) with tissue variance (Figure 1B). We performed this analysis for 81 ribosomal protein gene probesets for the 22 different tissues, with 3–5 replicates per tissue. Frequency distribution of the variance on the 1782 (81×22) sets of biologically replicated data is shown in Figure 1B. Median variance was 0.0055 and 95% of all data had a variance of 0.0463 or less. In contrast, differences in expression between tissues were much larger as median variance was 0.3011 and all variances were larger than 0.0463 (Figure 1B). The variance between tissues was significantly larger than that between replicates (Wilcoxon's rank sum statistic: p<0.0001). These data indicated that the variance of expression of ribosomal protein genes caused by replication within one tissue (both of biological and technical origin) was much smaller than the variation between tissues. We repeated this analysis with a smaller set of 15 genes described to be the most stably expressed [6]. Wilcoxon's rank sum statistic again resulted in a significant value (p<0.0001) meaning that also for these genes the variance between tissues was larger than between replicates. Similar significant data were obtained invariable of the normalization method used. These data indicated that the variation of expression of ribosomal protein genes caused by replication within one tissue was much smaller than the variation between tissues. Therefore, although most ribosomal protein genes could be called housekeeping genes, there was significant variation in expression across multiple tissues which indicated that these genes cannot be used in all conditions as reference genes.

In contrast to our data, a subset of ribosomal protein genes was described as stable over a large set of publicly available arrays [6]. The criterion for genes to be considered stable was their expression profiles showing a coefficient of variation (CV) <4% across all tissues (the CV is calculated as the standard deviation divided by the mean). The use of CV as a measure implies that the variance increases with higher expression levels. Our results however showed that the biological variation did not increase with higher expression levels, but rather the opposite (data not shown), invalidating the use of a CV cutoff as a stability criterion. Moreover, such a global criterion was incapable of accounting for smaller numbers of differing tissues. To illustrate, we calculated the following hypothetical example. Out of a total of 13629 samples (the same number as in the meta analysis), 100 samples were taken from a tissue in which a certain gene was only marginally expressed (log2 expression normally distributed with mean 8 and standard deviation 0.3) whereas in the other 13529 samples this gene was more abundant (log2 expression normally distributed with mean 12 and standard deviation 0.3). We generated 10000 random expression profiles that followed this scheme and calculated the CV. Distribution of the CV is shown in Figure S1. The mean±standard deviation was 3.80±0.02 and all randomly generated profiles had a CV lower than 4%. This example illustrates that the CV<4% criterion applied on a large dataset would still include genes which were expressed significantly lower in a subset of tissues and thus would still retain them as candidate reference genes.

### 2. Ribosomal protein genes are co-expressed in a tissue-specific manner

#### 2.1 Tissue-specific variation

**Figure 2:**
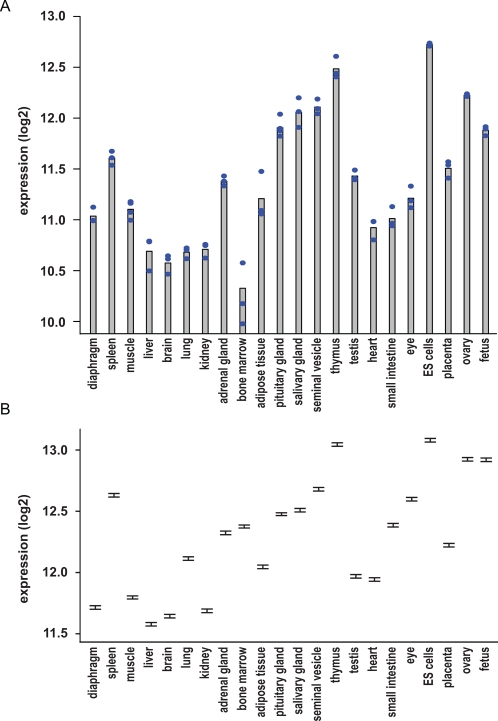
Tissue-specific co-expression of ribosomal protein genes. (A) Expression of *Rps13* in 22 different tissues. Individual measurements are displayed as blue dots and average corresponds to the top of the grey bars. Expression values are displayed in log2 scale. (B) Marginal means (estimated under the two-way ANOVA) of all ribosomal protein genes in each of the tissues, together with the 95 percent confidence interval.

We analyzed the origins of this variation across different tissues in our dataset. For *Rps13*, the most stably expressed gene [6], we obtained the graph displayed in Figure 2A. Large expression differences can be observed between different tissues, e.g. embryonic stem cells (ES cells) contained nearly 6-fold as much *Rps13* as compared to the brain cortex. One-way analysis of variance confirmed that these differences in expression were significant (F_21,48_ = 63.40, p<0.0001). This does not necessarily reflect the absolute levels of transcripts, since one tissue type might contain more RNA per cell as compared to another. Applying other normalization methods also revealed significant differences between tissues (data not shown). In addition, we analyzed expression of the human *Rps13* expression within the GDS596 record in Gene Expression Omnibus (GEO) which is a subset of the data used for the meta-analysis [6]. The expression profiles across the 79 physiologically normal human tissues in this dataset also displayed significant variation (F_78,79_ = 4.54, p<0.0001).

This difference in expression between tissues of *Rps13* was representative for most of the ribosomal proteins. Moreover, tissues in which *Rps13* was highly expressed had consistently higher expression levels for all ribosomal proteins. The occurrence of significant differences in expression between the tissues was already established in general by the tissue main effect in the two-way ANOVA. Next, we investigated between which specific tissues the expression levels differed, by means of pairwise comparisons of all possible tissue pairs using Tukey's multiple comparisons procedure, maintaining the overall significance level at .05. In Figure 2B, we display the estimated marginal means (estimated under the two-way ANOVA) together with the 95 percent simultaneous confidence intervals. It can be observed that many intervals do not overlap and, specifically for thymus, ES cells, ovary, and fetus there was no overlap with intervals of the other tissues.

In favor of a biological explanation underlying these differences between tissues, we noted that certain tissues consistently had a higher expression for the whole set of mRNA's encoding large and small subunit ribosomal proteins. Interestingly, such tissues contain either a high percentage of proliferating cells (ES cells, fetus, lymphoid tissues) and/or are specialized in exocrine protein secretion (salivary gland, seminal vesicle). Alternative to a biological explanation, one might argue that the high variance amongst tissues and tissue-specific co-regulated expression of all transcripts encoding ribosomal proteins reflects tissue-dependent artifacts of the normalization procedure or a microarray batch effect. Therefore, using the same microarray data, we performed a similar ANOVA analysis on another family of housekeeping genes, those encoding for the mitochondrial respiratory chain proteins. These data are displayed in Figure S2A. Similar to the ribosomal protein genes, significant tissue effects can be observed, but tissues with a high expression in respiratory chain proteins were not the same as those with a high expression of ribosomal proteins. Especially striated muscle tissues such as heart, gastrocnemius muscle and diaphragm, displayed the highest tissue-effect on respiratory chain gene expression. This distinct behavior of a group of genes represented on the same arrays excluded batch or normalization artifacts. Comparable to the data set obtained with the probes hybridizing to the ribosomal protein-encoding transcripts, the 69 respiratory chain proteins exhibited significantly more inter-tissue variance (median = 0.55) than technical variance (median = 0.01) (p<.0001 using Wilcoxon's rank sum test) (Figure S2B).

#### 2.2 Unfolding analysis

**Figure 3:**
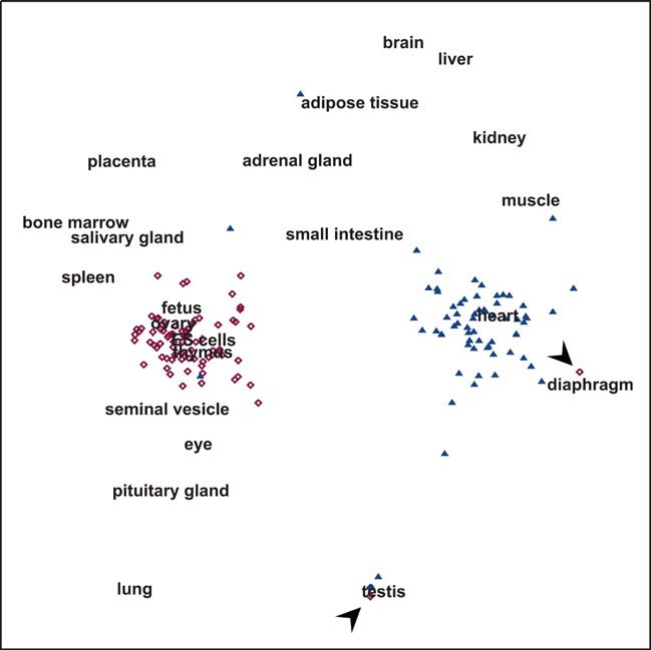
Multidimensional unfolding analysis of mRNA expression signals encoding 81 ribosomal proteins (purple symbols) versus 69 mitochondrial respiratory chain subunits (blue symbols). This analysis gives a graphical overview based on expression profiles; genes with a high expression in a certain tissue will be represented close to that tissue. Note that both groups of transcripts formed separate clusters. These clusters indicated high co-expression of ribosomal protein mRNA's in tissues that are active in exocrine protein secretion and/or cell division. Respiratory chain mRNA's were also co-expressed but were particularly high in striated muscle. Arrowheads indicate 2 ribosomal protein genes outside of the cluster. The upper arrowhead is *Rpl3l*, which clustered closely to the contractile tissues. The lower arrowhead is *Rpl39l*, located close to testis.

The tissue-specific expression of the ribosomal protein genes and the respiratory chain genes can also be supported by a purely exploratory (unsupervised, distribution free) analysis, being the multidimensional unfolding representation [8] depicted in Figure 3. The multidimensional unfolding model, which is an extension of multidimensional scaling to rectangular data [9], represents both the genes and the tissues as points in a low-dimensional space such that the (Euclidean) distances from a gene point to the tissue points reflect the expression profile. In other words, genes are located close to those tissues in which they are highly expressed (and far from those tissues in which they are barely expressed). Clearly, there were two distinct groups with the respiratory chain genes being located close to diaphragm, heart and muscle, and the ribosomal protein genes being located close to thymus, ES cells, ovary and fetus. Based on this consistent tissue-specific grouping of sets of housekeeping genes participating in separate pathways, a general normalization artifact to explain inter-tissue differences can be excluded.

An advantage of the unfolding representation (Figure 3) is that probe-specific behavior can be easily grasped and therefore a number of particularities can be observed, indicated by arrowheads. Two ribosomal protein genes were distant from their cluster, *Rpl3l* (ribosomal protein L3-like) and *Rpl39l* (ribosomal protein L39-like). Interestingly, *Rpl3l* could be found close to muscle, heart and diaphragm, exactly the group of tissues which had a very high expression of respiratory chain genes. An explanation for this exception to the clustered unfolding of ribosomal protein transcripts is proposed in section 3.1. *Rpl39l* was even more distinct on the unfolding plot and was in close proximity to testis; in multiple databases for different species (e.g. Genecards, Mouse Genome Informatics) this gene was indeed described to be testis-specific.

### 3. Origins of exceptionally deviating ribosomal protein mRNA expression profiles

#### 3.1 Tissue-specific isoforms: Rpl3 versus Rpl3l

On top of the gene and tissue effect contributing to expression variation, we described a significant gene-tissue interaction effect. This originated from individual genes displaying an aberrant expression in one or a few tissues. To illustrate, we further examined *Rpl3*, which has an isoform *Rpl3l* (*Rpl3*-like). The expression of *Rpl3* was significantly lower in skeletal muscle and diaphragm muscle –and to a lesser extent the heart- than in other tissues. However, exactly in these tissues, expression of *Rpl3*-like mRNA was specifically and abundantly detected. A similar result was observed independent of the way microarray data were processed (data not shown).

We also confirmed tissue-specific expression of *Rpl3l* in another species (rat), based on a more limited set of tissue microarrays (7 tissues, with 3 replicates for each tissue). Similar to mouse tissues, *Rpl3l* was exclusively expressed in muscle, the tissue with the lowest *Rpl3* expression (data not shown). In addition, inspection of publicly available microarray data from human tissues revealed a similar tissue specific expression. The GDS596 record in Gene Expression Omnibus (GEO) is based on data from 79 physiologically normal human tissues. In Figure S3A and S3B, the expression profiles of probesets 211073_x_at and 206768_at representing *Rpl3* and *Rpl3l* respectively is shown. Expression in heart and skeletal muscle was low for *Rpl3* and high for *Rpl3l* and vice versa for the other tissues. These additional data emphasize that –in addition to a general biological variation among tissues in ribosomal protein mRNA expression- evolutionary conserved more profound differences exist for *Rpl3*, related to expression of a tissue specific isoform, *Rpl3l* .

#### 3.2 Splice variants and alternative termination

**Figure 4:**
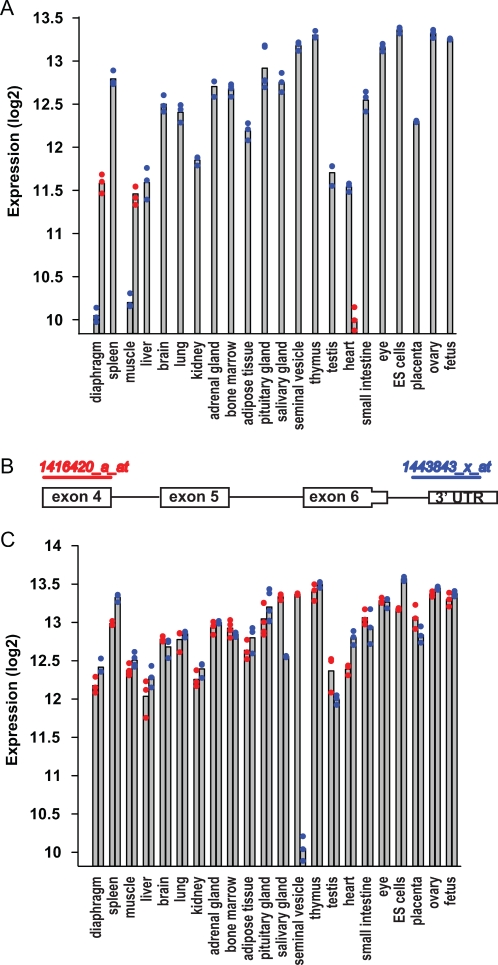
Two biological mechanisms underlying more pronounced variation in gene expression. (A) Expression of *Rpl3* (blue) and *Rpl3l* (red) in 22 different tissues. Note that tissues where *Rpl3l* was expressed (heart, diaphragm and muscle) had the lowest expression of *Rpl3*. (B) Schematic of Affymetrix probeset binding sites on the *Rpl9* transcript. Probeset 1443843_x_at (blue) binds most 3′ and probeset 1416420_a_at (red) binds more upstream. (C) Expression levels of *Rpl9* as detected by each of the probesets shown above. Both probesets yield similar signals, except in one tissue: seminal vesicles. This indicates that in seminal vesicles, an *Rpl9* transcript variant exists.

In addition to tissue-specific isoforms, profound differences in mRNA signal between tissues may also be the result of probe design and tissue-dependent alternative splicing or alternative termination. This was exemplified by ribosomal protein L9 (*Rpl9*), a gene ranked number 6 in the list of most stable genes [6]. Two Affymetrix mouse 430 2.0 probesets are targeted to exons of the mouse *Rpl9* gene, as shown on Figure 4B. Probeset 1416420_a_at is targeted to exon 4 (out of 7 exons) and probeset 1443843_x_at in part to an intron and exon 7 (3′ UTR). We plotted the tissue profile of hybridization signals for both probesets side by side (Figure 4C), with individual data points displayed as dots. Both probesets detected similar expression levels in all tissues, however, there was one strong exception as a very low signal was detected by probeset 1443843_x_at (blue) in seminal vesicles. For the same tissue, probeset 1416420_a_at (red) produced the highest signal (10-fold higher than 1443843_x_at). Again, this expression pattern could be observed independent of the way microarray data were processed (data not shown). Therefore, dependent on the region in the gene where the probes are binding to measure expression of a reference gene, profound differences among conditions can be the result of alternatively spliced or terminated transcript variants.

## Discussion

The selection of good reference genes is an ongoing debate. A confounding issue is the use of the terms “housekeeping” and “reference” genes; housekeeping genes are often used as reference genes, although for many of these individually it was shown they are not good reference genes. Some groups claimed good reference genes do not exist [3], whereas publications regularly appear in which new reference genes are proposed [10], [11]. Hsiao *et al.* studied gene expression in 19 different human tissue types [7] and defined a subset of 451 genes as housekeeping/maintenance genes based on a present call by the Affymetrix algorithm in all tissues. Most of the ribosomal protein encoding genes and some respiratory chain protein genes were included in this set. They noted that housekeeping genes define basic cellular processes and could be used as a reference standard but paradoxically mentioned that maintenance/housekeeping genes exhibit unique patterns for each specific tissue type. So far, there is still a discussion whether certain housekeeping genes can be used as reference genes over a broad range of tissues or conditions.

Recently, a large scale meta-analysis revealed a set of genes with an enhanced stability, of which the majority were ribosomal protein genes [6]. Interestingly, no ribosomal protein genes appeared in the list of stable housekeeping genes provided by Hsiao *et al*. In the present study, we further investigated the stability of ribosomal protein genes and identified some biological phenomena affecting this stability. By means of two-way ANOVA, we still observed a very significant difference in gene expression among different tissues, on top of any possible probe effect, which reflects the specific (e.g., metabolic, protein synthetic) needs of the different tissues. The variation which was attributable to technical replication errors and biological differences between animals was much smaller than the expression variation of these candidate reference genes between tissues. This indicated that ribosomal protein genes cannot be used as housekeeping reference genes when comparing different tissues. When we investigated the most stable gene, *Rps13*, we observed up to 6-fold expression differences across tissues in our dataset. These differences were representative for larger publicly available datasets. *Rps13* was highly expressed in exocrine protein secreting glands and tissues containing a high proportion of proliferating cells. In fact, we observed that almost all ribosomal protein genes were co-expressed at high levels in these same tissues. The most likely explanation for this observation is biological as a high need for ribosomes during protein secretion and cell division is expected. Actively growing mammalian cells contain 5 to 10 million ribosomes that must be synthesized each time the cell divides [12]. Proliferating lymphocytes, of which many are present in spleen and thymus, continuously produce cytoplasmic ribosomes [13]. This idea was further supported by molecular insights in the coordinated expression of genes required for ribosome biogenesis [14]. However, a second explanation for our data could be a series of tissue-specific artifacts, which resulted in tissue-dependent variations in hybridization signals, while the probed mRNA were -in fact- stably expressed. We provided strong evidence against this possibility by investigating in the same samples the expression of respiratory chain proteins, which are responsible for nutrient-induced ATP production and therefore essential for virtually all cells. When we quantified the nuclear encoded mRNAs for 69 of these subunits, we found again important variation between tissues in the expression level; importantly, the tissue pattern of expression levels was different as compared to ribosomal protein genes, which makes artifacts in sample preparation, hybridization or normalization unlikely. The tissue-specific behavior of gene groups was visualized by multidimensional unfolding analysis [8]. This method graphically plots genes close to the conditions (tissues) in which they have the highest expression. Strikingly, all ribosomal protein genes cluster in a group which is distinct from another housekeeping gene family, the respiratory chain genes. Two ribosomal protein genes were observed outside their cluster, *Rpl3l* and *Rpl39l*, which were the genes which had no present calls in certain tissues and were already known to be tissue-specific.

In further support of a biological explanation, we observed that these differences between tissues were not limited to one specific way of processing the microarray data. Gene expression measures are only obtained after a number of pre-processing steps which can be performed by different normalization procedures. We evaluated the effect of background corrected data, global scaling instead of quantile normalization and median polish summarization, but found all described effects present invariant of the processing method.

In addition to this general biological variation for all of these housekeeping genes, a more profound degree of variation seems based upon the existence of isoforms which are present only in a subset of tissues. This tissue specific expression of the isoform is often accompanied by a lowered expression of the other isoform(s). An example given here is ribosomal protein L3 (*Rpl3*), which has an isoform called ribosomal protein L3-like (*Rpl3l*). *Rpl3* was described to be ubiquitously expressed in all tissues, whereas *Rpl3l* was strongly expressed in skeletal muscle and heart tissue [15], exactly the tissues which had the lowest *Rpl3* expression. *Rpl3* expression is autoregulated by alternative splicing of overexpressed transcripts, followed by degradation of these transcripts through nonsense-mediated RNA decay [16]. Expression of *Rpl3l* in certain tissues might also favor alternative splicing of *Rpl3*, which would explain the lower expression in these tissues. Also for respiratory chain protein genes, tissue-specific isoforms have been described. The gene for the heart isoform of cytochrome c oxidase subunit VIa (*Cox6ah*) is expressed only in striated muscle, whereas the gene for the liver form (*Cox6al*) is expressed in all tissues, albeit at low levels in contractile muscle [17].

Another origin of variation in gene expression data relates to the probe position relative to the gene they are designed to interrogate. Since Affymetrix 3′ expression arrays contain probes designed at the 3′ end of genes, they may falsely not detect any transcript when the transcript is alternatively terminated before the binding site or when alternative splicing occurs. We showed evidence for alternative splicing or termination specifically in one tissue, being the seminal vesicle, where a shortened transcript was present. Seminal vesicle was not included in any of the 13629 arrays used for the data analysis by de Jonge *et al*. Even if this tissue would be represented, then still the alternative splicing or termination could be obscured by the averaging over multiple probesets. More importantly, when a single QPCR probe would be designed in this region, this effect might unexpectedly appear. Terminal changes may affect regulation by skipping (or introducing) microRNA binding sites or even cause differential subcellular localization. It is estimated that 40–79% of human genes with multiple exons produce transcript variants [18].

How can these differences in gene expression across many tissues, both in our own dataset and in public datasets be reconciled with the finding of stable housekeeping genes in a large meta-analysis [6]? One reason that de Jonge *et al.* still retain 16 stable genes (of which 15 are listed in the paper) is based on their selection criteria. A CV<4% is being used. In our example with the random expression profiles (data displayed in Fig S2), we calculated the effect of 100 tissues having a significantly lower expression of a gene in a set of 13629 arrays. We show that none of these examples had a CV higher than 4%. Obviously, when a large number of arrays are used, outliers will not easily influence this CV. Moreover, all of these examples also satisfy the two other criteria used by de Jonge *et al.*, showing that these criteria do not exclude potential reference genes which are significantly lower in a subset of tissues.

A more important reason that none of these examples would be excluded by the criteria used by de Jonge *et al.* is their interpretation of the maximum fold change (MFC). A ratio of 2 was applied to the log2 transformed expression values instead of the absolute expression values (see Table S1 in [6]). This results in a high range of expression values that can meet the criterion of de Jonge *et al*: the maximum expression that can be measured using Affymetrix chips is a log2 value of 16, which would result in log2 values above 8 still included as a potential reference gene, representing a 256 fold change in expression values.

In this paper, the reference gene status of ribosomal protein genes was questioned by showing that as a group they were more highly expressed in tissues with faster cell division and by showing more profound differences between conditions on the basis of tissue-specific isoforms or transcript variants. This supports the idea that even for housekeeping genes, whose products are indispensable for every living cell and which are relatively stably expressed, there are tissue-specific differences based upon extra demands in the required rate at which new housekeeping proteins need to be produced to maintain cell function. For a replicating cell, this means the extra synthesis of a new set of ribosomes, and for skeletal muscle, the maintenance of the mitochondrial respiratory chain to sustain ATP production for mechanical work. The selection of good reference genes will be thus be dependent on the subset of tissues used in a particular experiment and the experimental variables. As previously discussed, it seems unlikely to find genes which are expressed at the same level across all tissues of an organism. Therefore, we caution against using so-called stable genes identified by meta-analyses when designing an experiment. However, we support the use of microarrays to select reference genes, since this permits the selection of the most stable genes within the limited subset of tissues/conditions present in the particular experiment. The optimal set of reference genes depends on the tissue and should be selected and evaluated for each series of experiments [19]. This has already been successfully described by means of microarray [20], [21] or QPCR screens [19], [22]. The genes found through these screens may very well be ribosomal protein genes, but need to be verified for stable expression before use as a reference gene.

## Materials and Methods

### Preparation of tissues and purified cells

All experiments based upon laboratory animals were approved by committees for animal welfare at the Katholieke Universiteit Leuven. The following tissues were hand dissected from 10–12 week old C57Bl6 mice: liver, gastrocnemius muscle, brain, heart, adrenal gland, eye, small intestine, thymus, epidydimal adipose tissue, pituitary gland, kidney, parotis gland, spleen, lung, diaphragma, bone marrow, testis, and seminal vesicles (males); ovary and placenta (females). Fetal tissue was isolated at day 16. Embryonic stem cells were isolated as described in [23]. Tissues were rinsed in phosphate-buffered saline, frozen in liquid nitrogen and stored at −80°C.

### RNA extraction

Total RNA was extracted using TRIzol Reagent according to the manufacturer's protocol (Gibco BRL, Carlsbad, CA), followed by a cleanup procedure with RNeasy columns (Qiagen, Cologne, Germany). Total RNA from pituitary gland, adrenal gland and embryonic stem cells was extracted using the Absolutely RNA microprep from Stratagene (CA). The total RNA quantity and quality was determined using the NanoDrop ND-1000 spectrophotometer (NanoDrop Technologies, DW) and the 2100 Bioanalyzer (Agilent, Waldbronn, Germany), respectively. Total RNA profiles of all tested samples were similar with sharp 18S and 28S rRNA peaks on a flat baseline.

### mRNA expression analysis via microarray

Cellular mRNA was reverse transcribed into cDNA (SuperScript Choice System Invitrogen, Carlsbad, CA) using oligo-dT primers and a T7 RNA polymerase promoter site. Two µg of total RNA was used to prepare biotinylated cRNA with IVT labeling kit (Affymetrix, Santa Clara, CA) according to the Genechip expression analysis technical manual 701025 Rev.5, except for adrenal gland and pituitary gland where 1 µg of total RNA was used. The concentration of labeled cRNA was measured using the NanoDrop ND-1000 spectrophotometer. Labeled cRNA was fragmented in a fragmentation buffer during 35 min at 94°C. The quality of labeled and fragmented cRNA was analyzed using the Agilent bioanalyzer 2100. Fragmented cRNA was hybridised to mouse 430 2.0 arrays (Affymetrix) during 16 h at 45°C. The arrays were washed and stained in a fluidics station (Affymetrix) and scanned using the Affymetrix 3000 GeneScanner.

### Data Analysis

We used a microarray dataset consisting of 22 different murine tissues, with 3–5 replicates for each tissue (in total 70 microarrays). Quality controls of the arrays were according to manufacturer's criteria. All CEL files were analyzed using GCOS (Affymetrix GeneChip Operating Software) and the affy library [24] of the BioConductor project [25]. We independently applied the MAS5 algorithm using global scaling to 150 (to assess the present calls) and RMA. We performed RMA with and without convolution background adjustment and with either median polish or average difference summarization. Results shown are based on RMA data uncorrected for background, with probe-level quantile normalization, and average difference summarization. All data were log2 transformed for normalization and all further data analysis was performed on log2 transformed data. For each of the genes discussed where more than one probeset referred to the same gene annotation, only the best performing probeset with the highest expression values was used in order to avoid bias towards transcripts with more than one probeset. The data files have been deposited in the NCBI Gene Expression Omnibus (GEO, http://www.ncbi.nlm.nih.gov/geo/) and are accessible through GEO series accession number GSE9954.

### ANOVA

The analysis of variance was carried out using the generalized linear model (GLM) procedure in SAS. For the 81 one-way analyses of variance, significance was set equal to .01/81 = 0.00012 to keep the overall type I error at .01. The assumption of homoscedasticity using the Brown-Forsythe test, was met for all probes at the .01 level of significance and for all probes except one (*Rpl37a*) at the .05 level. For the two-way ANOVA, both the tissue and probe factor were treated as fixed (leading to fixed model) and options were specified to obtain sequential sum of squares. Since this was an unbalanced design, we used the type I (sequential) sum of squares considering the probe effect as the first effect. Note that in this analysis, independence at probe level was assumed. The pairwise comparisons, using Tukey's multiple comparison procedure were obtained on the estimated marginal means by use of the LSMEANS statement and appropriate options in this GLM procedure.

### Rank sum test

The Wilcoxon rank sum statistic was obtained from S-PLUS and used to test the null hypothesis of equal means versus the one-sided alternative that the mean variance between tissues was larger than the mean variance between replicates.

### Multidimensional unfolding

The average (over replicates) expression values, obtained from the log2 transformed data of 69 probesets for nuclear encoded respiratory chain genes and 81 probesets for ribosomal protein mRNA's, were submitted to the publicly available GENEFOLD toolbox [8], using a multistart procedure based on 100 semi-rational starts and convergence set to 1000 iterations or a difference in loss with the previous iteration smaller than 1e-5.

## Supporting Information

Figure S1 Calculated CV when out of a total of 13629 samples (the same number as in the meta analysis), 100 samples were taken from a tissue in which a certain gene was only marginally expressed (log2 expression normally distributed with mean 8 and standard deviation 0.3) whereas in the other 13529 samples this gene was abundant (log2 expression normally distributed with mean 12 and standard deviation 0.3). We generated 10000 random expression profiles that follow this scheme and calculated the CV. The mean±standard deviation was 3.80±0.02 and all randomly generated profiles had a CV lower than 4%.
(6.73 MB TIF)

Figure S2 (A) Marginal means (estimated under the two-way ANOVA) of expression values for mRNAs encoding 69 respiratory chain proteins in each of the tissues, together with the 95 percent confidence interval. (B) Variance of expression levels for respiratory chain genes within replicates of tissues, representing biological variation between animals and technical error on measurements (triangles) compared to variance of expression between tissues (circles). Variance within replicated measurements was significantly smaller than variance of expression between different conditions (p<.0001 using Wilcoxon's rank sum test).
(0.74 MB TIF)

Figure S3 Conservation in mammals of tissue specific expression of Rpl3 isoforms (A) GDS596 record for probeset 211073_x_at in GEO, showing Rpl3 expression across 79 physiologically normal human tissues. (B) GDS596 record for probeset 206768_at in GEO, showing Rpl3l expression across 79 physiologically normal human tissues. Expression in heart and skeletal muscle was low for Rpl3 and high for Rpl3l and vice versa for the other tissues.
(2.75 MB TIF)
