# Supplementary figures and images for: Using Ribosomal Protein Genes as Reference: A Tale of Caution
Source: PLoS One. 2008 Mar 26;3(3):e1854. doi: 10.1371/journal.pone.0001854 (PMC2267211; doi:10.1371/journal.pone.0001854)

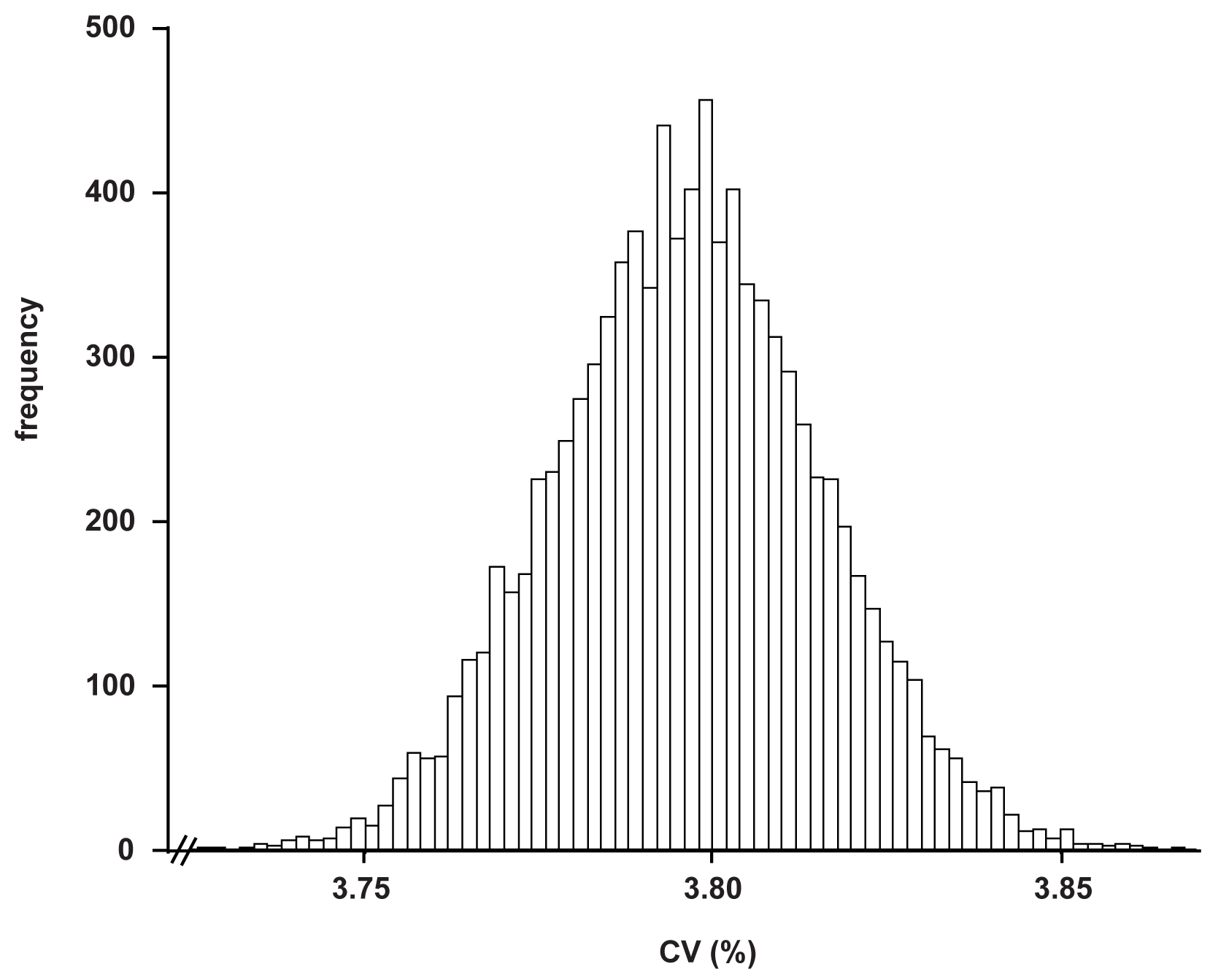

Supplement: Figure S1 — Calculated CV when out of a total of 13629 samples (the same number as in the meta analysis), 100 samples were taken from a tissue in which a certain gene was only marginally expressed (log2 expression normally distributed with mean 8 and standard deviation 0.3) whereas in the other 13529 samples this gene was abundant (log2 expression normally distributed with mean 12 and standard deviation 0.3). We generated 10000 random expression profiles that follow this scheme and calculated the CV. The mean±standard deviation was 3.80±0.02 and all randomly generated profiles had a CV lower than 4%. (6.73 MB TIF) [file pone.0001854.s001.tif]

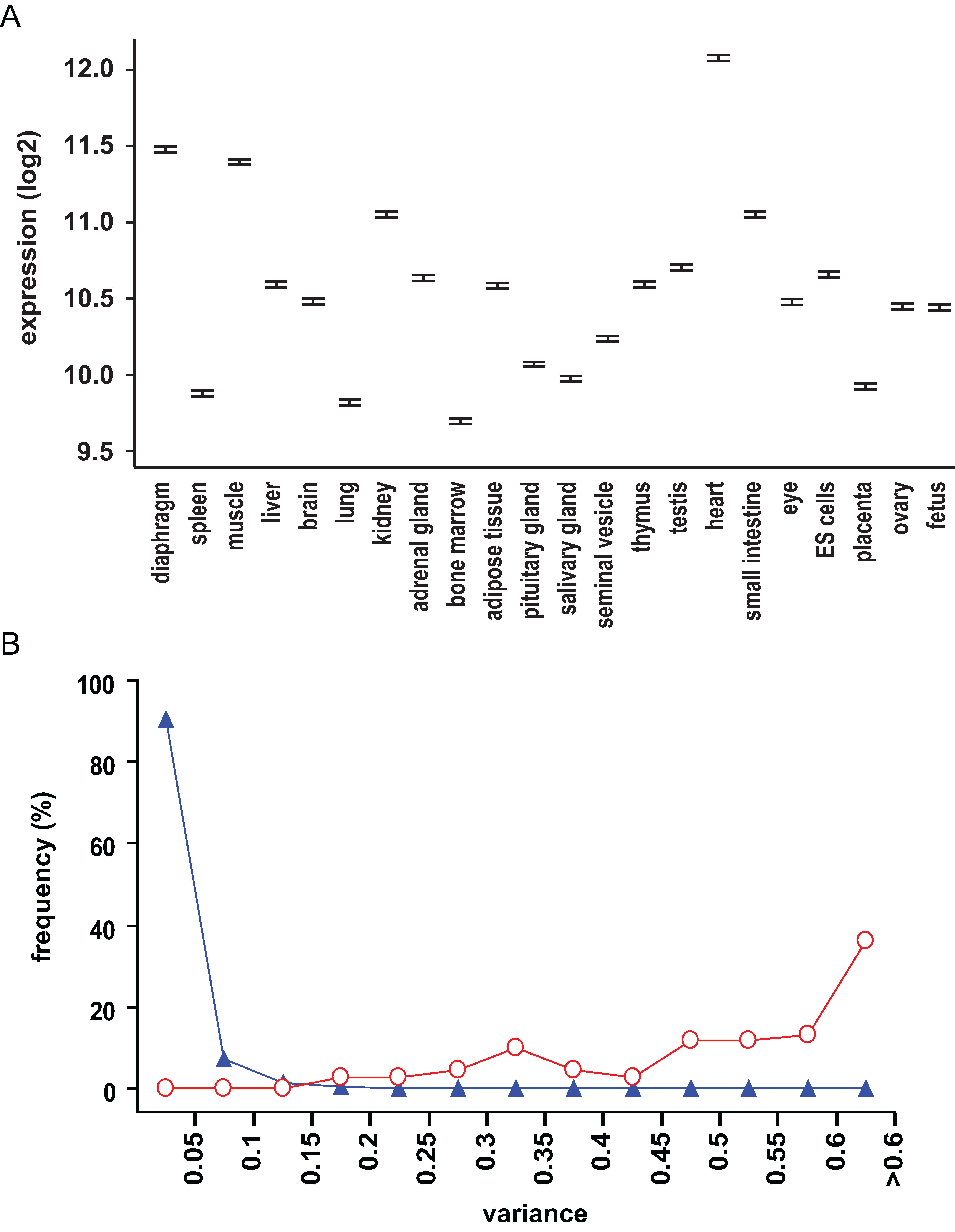

Supplement: Figure S2 — (A) Marginal means (estimated under the two-way ANOVA) of expression values for mRNAs encoding 69 respiratory chain proteins in each of the tissues, together with the 95 percent confidence interval. (B) Variance of expression levels for respiratory chain genes within replicates of tissues, representing biological variation between animals and technical error on measurements (triangles) compared to variance of expression between tissues (circles). Variance within replicated measurements was significantly smaller than variance of expression between different conditions (p<.0001 using Wilcoxon's rank sum test). (0.74 MB TIF) [file pone.0001854.s002.tif]

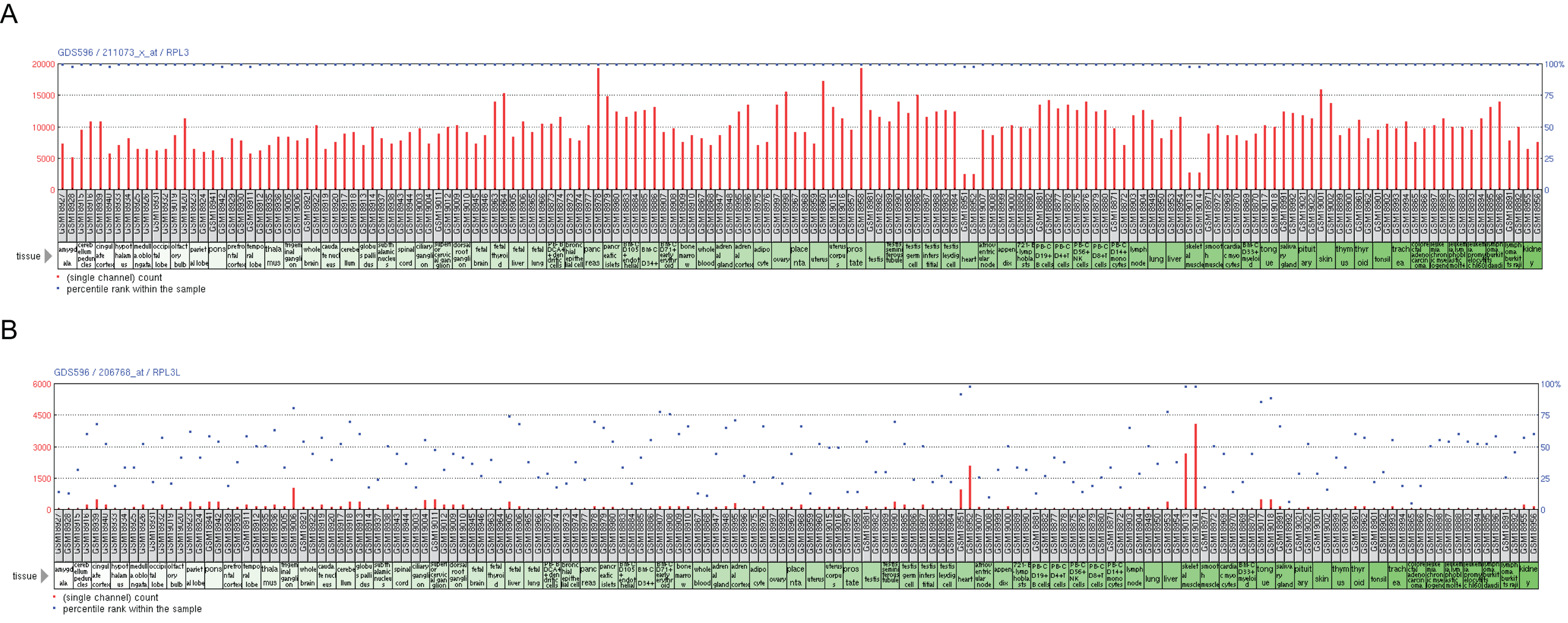

Supplement: Figure S3 — Conservation in mammals of tissue specific expression of Rpl3 isoforms (A) GDS596 record for probeset 211073_x_at in GEO, showing Rpl3 expression across 79 physiologically normal human tissues. (B) GDS596 record for probeset 206768_at in GEO, showing Rpl3l expression across 79 physiologically normal human tissues. Expression in heart and skeletal muscle was low for Rpl3 and high for Rpl3l and vice versa for the other tissues. (2.75 MB TIF) [file pone.0001854.s003.tif]
